# Assessing the perception of Parkinson’s disease in Al-Ahsa, Saudi Arabia among the visitors of a public campaign: before and after survey

**DOI:** 10.3389/fneur.2024.1365339

**Published:** 2024-04-03

**Authors:** Alia A. Alokley, Fatimah M. Alhubail, Abdullah M. Al Omair, Rawan A. Alturki, Rabab M. Alhaddad, Ali M. Al Mousa, Sarah A. Busbait, Mohammed A. Alnaim

**Affiliations:** ^1^Faculty of Neurology Department, College of Medicine, King Faisal University, Hofuf, Saudi Arabia; ^2^Department of Neurology, College of Medicine, King Faisal University, Al-Ahsa, Saudi Arabia

**Keywords:** Parkinson disease, public health awareness, neurodegenerative diseases, symptoms perception, management options, health campaign

## Abstract

**Introduction:**

Insufficient knowledge and beliefs hinder the early diagnosis and provision of adequate treatment and care for PD patients, causing socio-economic burdens. Raising public awareness and understanding the knowledge gap is crucial for effective educational programs and early detection. This study aims to assess the perception of Parkinson’s disease (PD) among visitors of a public campaign conducted to raise PD awareness and knowledge to facilitate early detection and management.

**Methods:**

A before-and-after study was conducted from May to June 2023, to assess the perception of PD among a public PD campaign visitors in Al-Ahsa, Saudi Arabia. The campaign included educational booths discussing PD symptoms, causes, diagnosis, management, and support. Participants completed a self-administered online questionnaire before and after the campaign. The data were analyzed using SPSS.

**Results:**

The study included 48 eligible individuals. The mean knowledge score was significantly enhanced following the campaign, rising from 12 to 17.77 points (*p* < 0.05). The symptoms of PD that showed a significant difference were slow movement, tremors, depression, memory problems, and sleep problems. The campaign had a beneficial effect on participants’ general understanding of PD.

**Discussion:**

The study showed that public awareness campaigns successfully raised community awareness of PD. Participants’ knowledge improved after the campaign, highlighting its positive effect. Further research could explore measuring the continuity of awareness over a longer period and its impact on improving patients’ lives and could expand the sample size.

## Introduction

Parkinson’s disease (PD) is the second most common neurodegenerative ailment after Alzheimer’s disease, affecting approximately 2–3% of the global population (1, 2). According to Al Rajeh et al. (3) a community survey study conducted in Saudi Arabia estimated the prevalence of PD to be 27 per 100,000 population. Moreover, a study implemented by Safiri et al. (4) demonstrating the prevalence of PD in the MENA region to be 82.6 per 100,000 population. PD is a complex age-related neurodegenerative disease associated with dopamine depletion leading to motor and nonmotor impairment. Loss of dopaminergic neurons in the midbrain’s substantia nigra pars compacta [SN] and the presence of Lewy bodies, cytoplasmic inclusions containing insoluble alpha-synuclein aggregates, are the pathological characteristics of PD (2, 5). PD risk is influenced by a variety of environmental and genetic factors, with various factors predominating in different patients (5).

Non-motor symptoms such as anosmia, constipation, depression, and REM sleep behavior disorder can appear years before motor deficits. Nevertheless, asymmetric resting tremor, cogwheel rigidity, and bradykinesia are the main diagnostic features. Later stages can involve autonomic dysfunction, pain, and cognitive impairment (2, 5). According to the initial presentation and patient’s age, the first-line management can be levodopa-carbidopa, dopamine agonists, anticholinergics, MAOIs, COMTIs, and amantadine. There are also interventional therapies available, including ultrasonic lesioning, and deep brain stimulation surgery, with cell replacement and gene therapy being a developing area (6).

Insufficient knowledge and beliefs hinder the early diagnosis and provision of adequate treatment and care for PD patients, causing socio-economic burdens. Raising public awareness and understanding the knowledge gap is crucial for effective educational programs and early detection (7–9). Low awareness combined with stigma related to chronic diseases, particularly PD, and the absence of resources in regional languages, such as Arabic, further compound the problem (2).

To combat this issue affecting the Alahsa population, a public awareness campaign has been conducted. However, the efficacy of such campaigns has not been thoroughly evaluated. Therefore, in this paper we aim to assess the overall level of knowledge about Parkinson’s disease among the visitors of this campaign and to evaluate its effectiveness in spreading awareness about Parkinson’s disease. Researchers expect that there will be an increased level of knowledge in the post-campaign survey, indicating that the message is conveyed correctly. This research has immense benefits for society as it can increase public knowledge about Parkinson’s disease, which might ultimately lead to early diagnosis and treatment.

## Methods

This before-and-after study was initiated in May 2023. A PD awareness campaign was held, in which several booths discussed the aspects of PD symptoms. These organized stations discussed the causes, signs and symptoms, diagnosis, management, and method to support patients with PD and their caregivers. A station was provided to simulate some PD symptoms for the visitors. The campaign was conducted in a mall located in Al-Ahsa city, Saudi Arabia, and lasted 3 days.

### Data collection

All visitors of the campaign were asked to fill in a self-administered online questionnaire made using Google Forms before going through the campaign. In the pre-campaign questionnaire, participants were asked for a phone number for two reasons. The first was to utilize the phone number as an identifier for the participants, to confirm that they had completed the pre- and post-campaign questionnaires. The other reason was to contact participants later to compare and evaluate the retention of knowledge. Data were obtained from the pre-campaign questionnaire during the three days of the campaign. Then, the participants were contacted one month later to complete the post-campaign questionnaire. Participants who had not completed either questionnaires, health care workers, or people under the age of 18 were excluded from the study. Data were collected by using a previously validated questionnaire by Alyamani et al. (7). The Arabic version of the questionnaire was tested using Cronbach’s alpha for reliability and validity with a score of 0.815. The questionnaire included three sections: informed consent, demographic data and relationship with PD, and general knowledge of PD.

### Statistical analysis

All the data were analyzed using Statistical Package for the Social Sciences (SPSS). Descriptive statistics were used to calculate frequencies. An inference analysis compared the different variables using a chi-square test and Fisher’s exact test as suitable. A *p*-value of 0.05 was considered as the cut-off point for the level of significance. To provide a numerical value from the used questionnaire, a score of 29 points was calculated for each participant; 1 point for each correct answer, and 0 points for each wrong or unknown answer. This score was used to calculate the means of overall knowledge throughout the study stages (pre- and post-). To evaluate the effect of the awareness campaign, a paired t-test was conducted to compare the means of the sample.

### Ethical approval

Participation in the study was voluntary. The purpose of the study and the expected time were reported, and online consent was obtained prior to completing the survey. Deanship of Scientific Research at King Faisal University was contacted for the ethical approval clearance for this study which was provided on May 4th, 2023 (KFU-REC-2023-MAY-ETHICS813).

## Results

A total of 82 participants who completed the electronic surveys were eligible based on the inclusion criteria. Of them, 24 subjects did not complete the post-campaign questionnaire when contacted, 10 reported wrong numbers and they did not participate in the study. Subsequently, all the previously mentioned 34 subjects were excluded from the study. The final included sample was 48 eligible subjects.

Participants in both stages of the study showed a minimum score of 1 point and a maximum of 27 points out of 29 with regard to their overall knowledge of PD. The mean score of the sample was 12 points ± 7.57 for the responses reported before attending the booths of the campaign, whereas 17.77 points ± 5.44 after receiving the educational information that was provided in each booth. A paired t-test analysis showed a −5.77 mean with a significant *p*-value of 0.00001 (*p* < 0.05) between the means difference of the sample (before and after). This finding indicates a significant improvement in the mean score of overall knowledge, after attending the campaign.

With regard to the sample characteristics, more than half of the participants were aged between 18–30 years 56.3%, and the remaining were aged between 31–49 years of age (43.8%). Similarly, the gender distribution of the sample was 56.3% for females, and 43.8% for males. Furthermore, almost all subjects had educational degrees from secondary school or higher, with bachelor’s holders forming approximately half of the total sample (52.1%). Whereas only three participants had a middle school educational degree and only one subject held an elementary school degree. Most of the participants had no connection to PD, nonetheless, two individuals had a family member with PD, one had a friend with PD, and only one individual was diagnosed with PD. Further details are shown in Table 1.

**Table 1:** Demographic data.

Item	Category	*N*	%
Age	18–30	27	56.3
	31–49	21	43.8
Gender	Male	21	43.8
	Female	27	56.3
Educational degree	Elementary school	1	2.1
	Middle school	3	6.3
	Secondary school	12	25.0
	Diploma	7	14.6
	Bachelors	25	52.1
Do you have a connection to Parkinson’s disease?	I have Parkinson’s disease	1	2.1
	I have a family member with Parkinson’s disease	2	4.2
	I have a friend with Parkinson’s disease	1	2.1
	I have no connection with Parkinson’s disease	44	91.7

**Figure 1:**
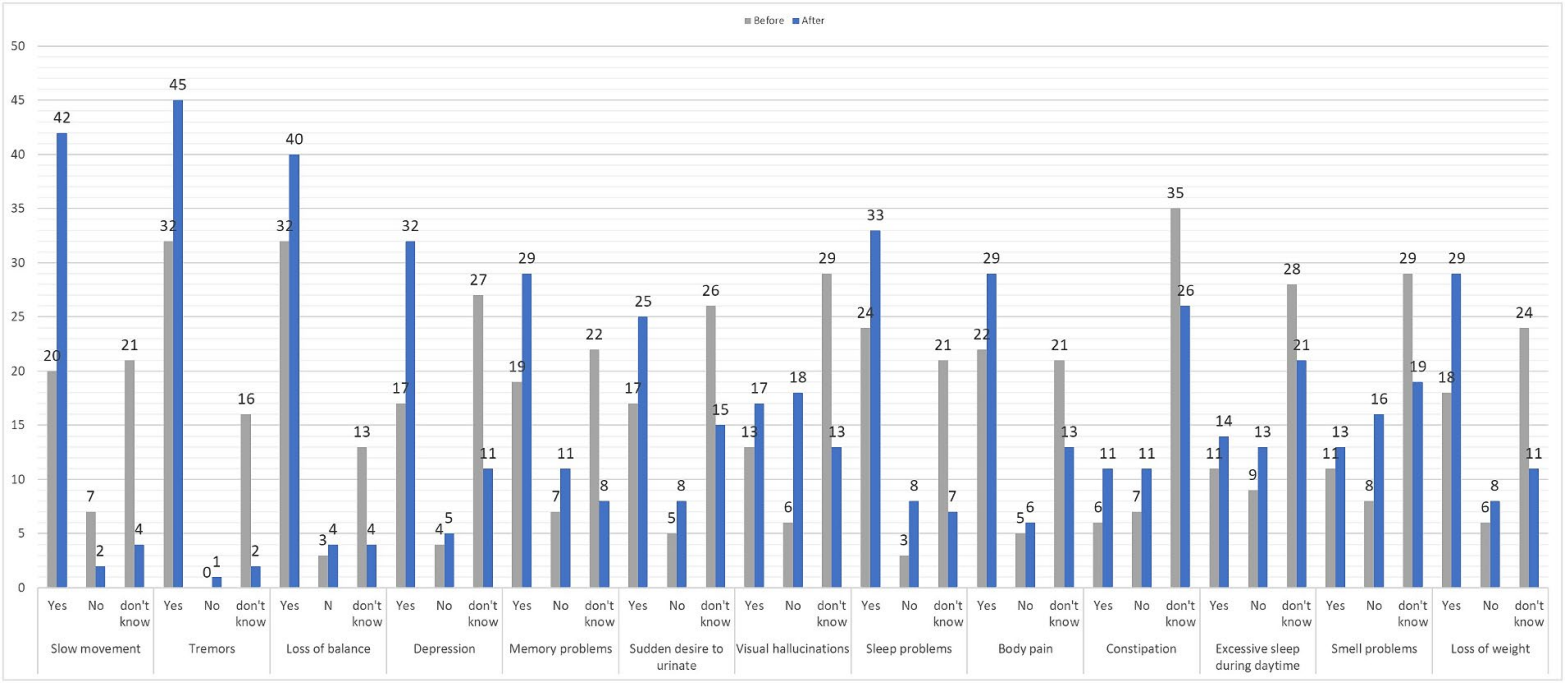
Knowledge of Parkinson’s disease symptoms.

The participant’s responses with regard to the knowledge of PD symptoms during the stages of the study are illustrated in Figure 1. The most correctly recognized PD symptoms before receiving the educational information were tremors and loss of balance (32 subjects), which; respectively, have increased to 45, and 40 subjects after going through the campaign booths. Followed by sleep problems, which were reported by 24 participants in the pre-campaign questionnaire, and 33 in the post-campaign questionnaire. However, the least reported symptoms in the pre-campaign questionnaire were constipation (6 subjects), and excessive daytime sleep (11 subjects). Similarly, they were the least reported in the post-campaign questionnaire (11 and 14 subjects, respectively). Furthermore; symptoms that showed a significant difference between the two stages of the study included slow movement, tremors, depression, memory problems, visual hallucinations, sleep problems, and weight loss (*p* < 0.05).

With respect to the participant’s knowledge regarding general information about PD, the results are shown in Table 2. The majority of the participants were aware that patients with PD seek treatment from the neurology specialty even before receiving any educational lessons (29 subjects), and this awareness increased to 32 subjects after receiving information. However, although incorrect options were chosen less in the post-campaign questionnaire, the number of people reporting neurosurgery received more responses on the post-campaign questionnaire. Furthermore, in the pre-campaign questionnaire, the majority of participants were knowledgeable about available treatments options for PD (33 subjects), while almost only half of the sample were familiar with the association between PD and low dopamine levels (20 subjects), head injury and trauma (24 subjects), various incidences of patients in different age groups (20 subjects), and the provision of support to those who are affected, promoted by the Saudi Parkinson’s Disease Society. Moreover, subjects lacked knowledge of the distinction between PD and Alzheimer’s disease (8 subjects), the connection between PD, genetics, and social isolation (14 subjects), drug use and stroke (16 subjects), identifying age as an important risk factor, affection of multiple family members, and the appearance of tremors in all patients suffering from Parkinson’s disease (19 subjects).

**Table 2:** Knowledge of general information regarding Parkinson’s disease.

Question	Response	Before *N* (%)	After *N* (%)	*p*-value
Which specialty should patients with Parkinson’s disease seek treatment from?	Internal medicine	1 (2.08%)	0 (0%)	0.659^a^
	General surgery	1 (2.08%)	0 (0%)	
	Neurology^b^	29 (60.42%)	32 (66.67%)	
	Neurosurgery	8 (16.67%)	11 (22.92%)	
	Psychiatry	3 (6.25%)	2 (4.17%)	
	Family medicine	6 (12.5%)	3 (6.25%)	
Which types of treatment are available for Parkinson’s disease	Drug therapy	3 (6.25%)	7 (14.58%)	0.180^a^
	Surgical treatment	2 (4.17%)	1 (2.08%)	
	Occupational and functional therapy	10 (20.83%)	4 (8.33%)	
	All the above^b^	33 (68.75%)	36 (75%)	
Parkinson’s disease and Alzheimer are different names for the same disease* (Correct answer is “FALSE”)	True	8 (16.67%)	8 (16.67%)	0.001
	False^b^	18 (37.5%)	34 (70.83%)	
	I do not know	22 (45.83%)	6 (12.5%)	
Parkinson’s disease is associated with a lysis of one of the brain tracts	True^b^	25 (52.08%)	34 (70.83%)	0.160^a^
	False	2 (4.17%)	1 (2.08%)	
	I do not know	21 (43.75%)	13 (27.08%)	
Parkinson’s disease is associated with low dopamine levels (Neurotransmitter)*	True^b^	20 (41.67%)	35 (72.92%)	0.003^a^
	False	2 (4.17%)	2 (4.17%)	
	I do not know	26 (54.17%)	11 (22.92%)	
Parkinson’s disease can be associated with genetics*	True^b^	14 (29.17%)	23 (47.92%)	0.009
	False	6 (12.5%)	12 (25%)	
	I do not know	28 (58.33%)	13 (27.08%)	
Parkinson’s disease can be related to head injuries and trauma	True^b^	24 (50%)	31 (64.58%)	0.072
	False	6 (12.5%)	9 (18.75%)	
	I do not know	18 (37.5%)	8 (16.67%)	
Parkinson’s disease can be related to drug use*	True^b^	17 (35.42%)	18 (37.5%)	0.047
	False	7 (14.58%)	16 (33.33%)	
	I do not know	24 (50%)	14 (29.17%)	
Parkinson’s disease can be related to strokes*	True^b^	16 (33.33%)	28 (58.33%)	0.011
	False	7 (14.58%)	9 (18.75%)	
	I do not know	25 (52.08%)	11 (22.92%)	
Age is an important risk factor of Parkinson’s disease*	True^b^	19 (39.58%)	31 (64.58%)	0.002
	False	5 (10.42%)	9 (18.75%)	
	I do not know	24 (50%)	8 (16.67%)	
All patients with Parkinson’s disease suffer from tremors* (Correct answer is “FALSE”)	True	19 (39.58%)	32 (66.67%)	0.001^a^
	False^b^	10 (20.83%)	12 (25%)	
	I do not know	19 (39.58%)	4 (8.33%)	
Parkinson’s disease and parkinsonism are more common in elderly patients but can affect young patients also*	True^b^	20 (41.67%)	41 (85.42%)	0.00001^a^
	False	5 (10.42%)	2 (4.17%)	
	I do not know	23 (47.92%)	5 (10.42%)	
Parkinson’s disease can affect more than one member of the family*	True^b^	19 (39.58%)	29 (60.42%)	0.039
	False	6 (12.5%)	8 (16.67%)	
	I do not know	23 (47.92%)	11 (22.92%)	
There are drugs that can improve Parkinson’s disease symptoms*	True^b^	27 (56.25%)	37 (77.08%)	0.001^a^
	False	1 (2.08%)	6 (12.5%)	
	I do not know	20 (41.67%)	5 (10.42%)	
Parkinson’s disease patients are socially isolated	True^b^	14 (29.17%)	19 (39.58%)	0.145
	False	16 (33.33%)	20 (41.67%)	
	I do not know	18 (37.5%)	9 (18.75%)	
Parkinson’s disease patients and their families have support from the Saudi Parkinson’s disease Society*	True^b^	20 (41.67%)	34 (70.83%)	0.012^a^
	False	3 (6.25%)	1 (2.08%)	
	I do not know	25 (52.08%)	13 (27.08%)	

*Significant (*p* < 0.05).

a Fisher exact test was used when the cell counts were less than five.

b Correct Answer.

A statistically significant difference was reported between most of the investigated elements (11 out of 16) and the study stages (before and after) (*p* < 0.05), excluding knowledge about which specialty to seek for patients with PD, types of treatments available, the association between PD and lysis of one of the brain tracts, head injuries and trauma and social isolation.

## Discussion

To the best of our knowledge, this is the first study to measure awareness of PD while simultaneously highlighting the impact of campaigns conducted among the general population on altering the knowledge about the disease. Several studies have estimated the awareness level of PD in Saudi Arabia, Korea, Uganda, and Central Asia (7–10). However, none of the studied assumed a pre-post study design similar to that used in this research. In addition, further efforts are necessary, as research shows the need for stronger campaigns to deliver accurate information about PD, with a focus on reaching under-informed groups (9).

Research into PD knowledge and attitudes in Saudi Arabia has revealed that while public knowledge of PD is generally adequate, there are still common misperceptions about the condition, revealing a significant gap in knowledge about PD that must be addressed. Improving public awareness of PD and educating individuals about its symptoms and treatment requires the implementation of effective educational campaigns (7).

Before attending the campaign, the average score of participants was 12 points ± 7.57, and it significantly increased to 17.77 points ± 5.44. Success can be seen in previous campaigns, such as palliative care, premarital examination, and crown health, which significantly increased awareness rates by 62%, doubled results, and even achieved 11-fold increases, respectively (11–16). This emphasizes that public campaigns can help reconstruct misconceptions and ideas adopted by the general population to facilitate better overall knowledge that can hopefully impact the care and quality of life for PD patients. Recognition of the disease and seeking medical attention with the onset of earlier symptoms can alleviate motor and nonmotor signs, enhance physical function, and reduce disability (17).

The most correctly recognized PD symptoms before receiving the educational information were tremors and loss of balance. Similarly, Alyamani et al., Kaddumukasa et al., Tan et al., and Youn et al. found tremor to be the most recognized symptom among the general population (7–9, 12). This was a predicted result, because tremor is one of the cardinal motor symptoms in PD. Yet, the campaign did not correct the misperception that tremor is a mandatory feature in PD patients among the visitors as shown in Table 2. The presence of non-motor symptoms was highly unknown among many participants before the campaign. This was in consensus with the studies conducted in Riyadh and Asia, as non-motor symptoms were also underreported. Interestingly, even among PD patients, reporting non-motor symptoms have a level of neglect (7, 9). This is concerning because PD patients have a 6-fold increased risk of dementia (18). Fortunately, following booth attendance, depression, memory problems, visual hallucinations, sleep problems, and weight loss showed a statistically significant improvement in the rate of their recognition.

Regarding the general information about PD, we were able to detect a significant improvement in knowledge post-campaign reflected in 11 of 16 elements. One element that most participants were aware of was the appropriate physicians from whom to seek medical treatment, which were neurologists. While this is a positive finding, as neurologists are the mainstay health physician in treating PD, it is important to emphasize that PD requires a multidisciplinary team of physical, occupational, and speech therapists alongside the treating neurologists to establish the most optimal results PD patients (19). Unfortunately, a scarce number of centers provide such comprehensive care for PD patients in the MENA region (20). This calls for a crucial advancement, as this method of care is needed to improve the quality of life of PD patients.

### Limitation

A limitation that has been noticed is that the study sample size is small in proportion to the total number of campaign visitors, which was estimated to be around a thousand visitors. This was due to the fact that the greater majority of visitors did not commit to completing both questionnaires. This might raise the issue of nonresponse bias. In addition, this study did not include active surveillance on participants during filling of the post-test questionnaire, which would leave the possibility of subjects looking up for answers uncontrolled. Furthermore, although raising awareness can result into an environment where change is encouraged to be implemented. This study does not necessarily provide a reflection of the participants attitude in screening or seeking help for their elderly relatives.

## Conclusion and recommendation

Our study provided a comprehensive overview of the impact of volunteer campaigns in increasing community awareness about PD. The data results showed that most of the answers given by the study participants improved after passing through the booths compared to their knowledge before passing. This gives positive results to the extent of the Parkinson’s campaign’s impact on community awareness. Therefore, the responsibility becomes greater for healthcare workers to exert greater efforts in the field of medical campaigns. Further research could delve into measuring the continuity of awareness over a longer period and its impact on improving the lives of Parkinson’s patients, as well as expanding the number of study participants.

## Data availability statement

The raw data supporting the conclusions of this article will be made available by the authors, without undue reservation.

## Ethics statement

The studies involving human participants were reviewed and approved by the Deanship of Scientific Research at King Faisal University (KFU-REC-2023-MAY-ETHICS813). Written informed consent to participate in this study was provided by the patient/participants or patient/participants’ legal guardian/next of kin.

## Author contributions

AAA: Conceptualization, Supervision, Writing – original draft, Writing – review & editing. FA: Project administration, Supervision, Validation, Writing – original draft, Writing – review & editing. AMAO: Conceptualization, Project administration, Supervision, Writing – original draft, Writing – review & editing. RAA: Conceptualization, Writing – original draft, Writing – review & editing. RMA: Conceptualization, Data curation, Investigation, Writing – original draft, Writing – review & editing. AMAM: Data curation, Formal analysis, Methodology, Writing – original draft, Writing – review & editing. SB: Methodology, Visualization, Writing – original draft, Writing – review & editing. MA: Conceptualization, Investigation, Writing – original draft, Writing – review & editing.
